# Application of the Elitist-Mutated PSO and an Improved GSA to Estimate Parameters of Linear and Nonlinear Muskingum Flood Routing Models

**DOI:** 10.1371/journal.pone.0147338

**Published:** 2016-01-19

**Authors:** Ling Kang, Song Zhang

**Affiliations:** School of Hydropower and Information Engineering, Huazhong University of Science and Technology, Wuhan, China; Beihang University, CHINA

## Abstract

Heuristic search algorithms, which are characterized by faster convergence rates and can obtain better solutions than the traditional mathematical methods, are extensively used in engineering optimizations. In this paper, a newly developed elitist-mutated particle swarm optimization (EMPSO) technique and an improved gravitational search algorithm (IGSA) are successively applied to parameter estimation problems of Muskingum flood routing models. First, the global optimization performance of the EMPSO and IGSA are validated by nine standard benchmark functions. Then, to further analyse the applicability of the EMPSO and IGSA for various forms of Muskingum models, three typical structures are considered: the basic two-parameter linear Muskingum model (LMM), a three-parameter nonlinear Muskingum model (NLMM) and a four-parameter nonlinear Muskingum model which incorporates the lateral flow (NLMM-L). The problems are formulated as optimization procedures to minimize the sum of the squared deviations (SSQ) or the sum of the absolute deviations (SAD) between the observed and the estimated outflows. Comparative results of the selected numerical cases (Case 1–3) show that the EMPSO and IGSA not only rapidly converge but also obtain the same best optimal parameter vector in every run. The EMPSO and IGSA exhibit superior robustness and provide two efficient alternative approaches that can be confidently employed to estimate the parameters of both linear and nonlinear Muskingum models in engineering applications.

## Introduction

Accurate forecasting of flood wave movement in natural river channels is extremely important for the real-time monitoring, alert and control of floods, which are effective non-engineering measures for preventing tremendous loss of lives and property. Two categories of approaches for flood routing exist: hydraulic and hydrologic methods [1]. The former routes flood by numerically solving the famous Saint-Venant equations, which usually has strict requirements for the topographical data of the investigated stream channel (such as channel cross-section and roughness) and complicated computations [2]. Conversely, the latter is based on the continuity and empirical storage equations and is more widely used in engineering applications due to its simplicity. The Muskingum flood routing model, developed by McCarthy [3], is the most frequently applied hydrologic technique.

As is known to all, the precise estimation of parameters is the key point in applying the Muskingum method for real-time flood forecasting [4]. This problem is always formulated and solved by determining the values of Muskingum parameters using historical inflow-outflow hydrograph data based on a specified optimization criterion (i.e., optimization objective). During the past decades, two types of diverse techniques have been developed to deal with the problem: traditional mathematical methods and heuristic optimization algorithms. The mathematical methods include the least-squares method (LSM) [5], the Hooke-Jeeves (HJ) pattern search in conjunction with the linear regression (HJ+LR), the conjugate gradient (HJ+CG) or the Davidon-Fletcher-Powell (HJ+DFP) algorithms [6], the nonlinear least-squares regression (NONLR) [7], the Broyden-Fletcher-Goldfarb-Shanno algorithm (BFGS) [8] and the Nelder-Mead simplex (NMS) algorithm [9]. However, most mathematical methods mentioned above inevitably have some drawbacks, such as special derivation conditions, a time-consuming quality or initial parameter assumptions. Therefore, numerous researchers focus on heuristic optimization algorithms that are characterized by fast convergence and the ability to obtain better solutions in recent decades, such as the harmony search (HS) [10], the genetic algorithm (GA) [11], the standard, improved or hybrid particle swarm optimization algorithms (PSOs) [12–15], the immune clonal selection algorithm (ICSA) [16], the differential evolution (DE) [17], and the cuckoo search (CS) algorithm [18].

The purpose of this research is to apply the newly developed elitist-mutated PSO (EMPSO) algorithm [19] and an improved gravitational search algorithm (IGSA) to solve parameter estimation problems of different forms of Muskingum models (one linear structure and two nonlinear structures). The proposed IGSA is based on the gravitational search algorithm (GSA) [20]. These two improved algorithms both have no previous applications for such issues. In the IGSA, a modified velocity updating rule and the elite strategy are introduced to enhance the global search ability and accelerate the convergence speed of the basic GSA, respectively. The experimental results of 9 widely-used standard benchmark functions with diverse properties demonstrate the global optimization abilities of the EMPSO and IGSA. The application cases verify their validity and advantages in handling parameter estimation problems of both linear and nonlinear Muskingum models.

The remainder of this paper is organized as follows: In Sect. 2, we provide the structures and the flood routing procedures of three important linear and nonlinear Muskingum models, whose structure complexities increase with the number of parameters from two to four. In Sect. 3, we briefly describe the newly developed EMPSO and the IGSA presented in this study, and then they are tested on 9 minimization benchmark functions. In Sect. 4, the EMPSO and the IGSA are successfully applied in numerical cases (three typical flood events). The results and analysis are also presented in this section. We discuss some conclusions of our research work in Sect. 5.

## Muskingum Models

In previous decades, various forms of Muskingum models have been investigated [5, 18, 21]. Three typical linear or nonlinear Muskingum models and their corresponding flood routing equations or procedures are briefly described in this section: the original two-parameter linear Muskingum model (LMM) [3], a three-parameter nonlinear Muskingum model (NLMM) [5] and a four-parameter nonlinear Muskingum model that incorporates the lateral flow (NLMM-L) [18].

### LMM

The original LMM, which is based on the basic hypothesis that the storage within a river reach is a weighted function of inflow and outflow rates, employs the following continuity and storage equations.

$$\frac{dS_{t}}{dt}=I_{t}-O_{t}$$ (1)

$$\begin{array}{cc} S_{t}=K\left[xI_{t}+\left(1-x\right)O_{t}\right], & \left(\text{LMM}\right) \end{array}$$ (2)

where *S*_*t*_ = channel storage at time *t*; *I*_*t*_ and *O*_*t*_ = observed rates of inflow and outflow at time *t*, respectively; *K* = storage-time constant, which has a value that is similar to the flow travel time through the routing river reach; *x* = weighting factor, *x* ∈ (0, 0.3] for stream channels and *x* ∈ (0, 0.5] for reservoir storage. The finite difference solution for Eqs (1) and (2) and the flood routing procedure of LMM is given by Eqs (3)–(5).

$${\overset{\wedge }{O}}_{0}=O_{0}$$ (3)

$$\begin{array}{cc} {\overset{\wedge }{O}}_{t}=C_{0}I_{t}+C_{1}I_{t-1}+C_{2}{\overset{\wedge }{O}}_{t-1}, & \left(t=1,\ldots ,T\right) \end{array}$$ (4)

$$C_{0}+C_{1}+C_{2}=1$$ (5)

where ${\overset{\wedge }{O}}_{t}$ = estimated outflow at time *t*; *T* = total number of time intervals; *C*_0_, *C*_1_ and *C*_2_ = three coefficients of LMM. Note that the LMM is a two-parameter (*C*_0_, *C*_1_) Muskingum model because *C*_2_ = 1 − *C*_0_ − *C*_1_.

### NLMM

However, the relationship between the channel storage *S*_*t*_ and the weighted flow [*xI*_*t*_ + (1 − *x*)*O*_*t*_] is not always and essentially linear in many river reaches; thus, the use of LMM may be inappropriate. Hence, an additional exponent parameter *m* was introduced to consider the effect of nonlinearity. The following form of nonlinear Muskingum model has been suggested [5].

$$\begin{array}{cc} S_{t}=K{\left[xI_{t}+\left(1-x\right)O_{t}\right]}^{m}, & \left(\text{NLMM}\right) \end{array}$$ (6)

As shown in Eq (6), the NLMM is a three-parameter (*K*, *x* and *m*) Muskingum model and the LMM is a particular form of NLMM with *m* = 1. The rate of outflow *O*_*t*_ can be calculated by rearranging Eq (6):

$$O_{t}=\left(\frac{1}{1-x}\right){\left(\frac{S_{t}}{K}\right)}^{1/m}-\left(\frac{x}{1-x}\right)I_{t}$$ (7)

### NLMM-L

The LMM and NLMM are frequently viewed and discussed in the literature. However, they all disregard the lateral flow along the investigated reach despite the fact that lateral flow exists along many river reaches in actual flood events. Assuming that the lateral flow (*Q*_lat_) linearly varies along a river reach and can be expressed as a ratio (*α*) of the inflow rate (*Q*_lat_ = *αI*), O'Donnell [21] proposed another linear Muskingum model that consider lateral flow in 1985, it is expressed as Eqs (8) and (9).

$$\frac{dS_{t}}{dt}=I_{t}+Q_{\text{lat},t}-O_{t}=\left(1+\alpha \right)I_{t}-O_{t}$$ (8)

$$\begin{array}{cc} S_{t}=K\left[x\left(1+\alpha \right)I_{t}+\left(1-x\right)O_{t}\right], & \left(\text{LMM-L}\right) \end{array}$$ (9)

Inspired by the above assumptions by O'Donnell [21], in 2014, Karahan and Gurarslan [18] proposed a new nonlinear Muskingum model that takes the lateral flow into consideration after the integration of continuity Eq (8) and the NLMM in Eq (6):

$$\begin{array}{cc} S_{t}=K{\left[x\left(1+\alpha \right)I_{t}+\left(1-x\right)O_{t}\right]}^{m}, & \left(\text{NLMM-L}\right) \end{array}$$ (10)

As expressed in Eq (10), the NLMM-L is a four-parameter (*K*, *x*, *m* and *α*) Muskingum model. By rearranging Eq (10), the rate of outflow *O*_*t*_ can be calculated using Eq (11).

$$O_{t}=\left(\frac{1}{1-x}\right){\left(\frac{S_{t}}{K}\right)}^{1/m}-\left[\frac{x\left(1+\alpha \right)}{1-x}\right]I_{t}$$ (11)

### Routing Procedures of the NLMM and NLMM-L

In contrast to the LMM, the flood routing procedures of nonlinear Muskingum models NLMM and NLMM-L are highly complex. The routing procedures for the NLMM and the NLMM-L can be standardized using the following steps [2, 8, 18]:

**Step 1**: Assume values of the Muskingum parameters (*K*, *x* and *m* for NLMM; *K*, *x*, *m* and *α* for NLMM-L).
**Step 2**: Calculate the storage amount (*S*_*t*_) using Eq (6) for the NLMM and Eq (10) for the NLMM-L.
**Step 3**: After combining the continuity Eqs (1) and (8) with the corresponding outflow calculations in Eqs (7) and (11), the time rate of the storage change can be calculated using Eq (12) for the NLMM and Eq (13) for the NLMM-L.
  $$\frac{\Delta S_{t}}{\Delta t}=I_{t}-O_{t}=-\left(\frac{1}{1-x}\right){\left(\frac{S_{t}}{K}\right)}^{1/m}+\left(\frac{1}{1-x}\right)I_{t}$$ (12)
  $$\frac{\Delta S_{t}}{\Delta t}=\left(1+\alpha \right)I_{t}-O_{t}=-\left(\frac{1}{1-x}\right){\left(\frac{S_{t}}{K}\right)}^{1/m}+\left(\frac{1+\alpha }{1-x}\right)I_{t}$$ (13)
**Step 4**: Calculate the next storage using Eq (14), where Δ*t* is assumed to represent the unit time.
  $$S_{t+1}=S_{t}+\Delta S_{t}$$ (14)
**Step 5**: Calculate the next estimated outflow $\left({\overset{\wedge }{O}}_{t+1}\right)$ using Eq (15) for the NLMM and Eq (16) for the NLMM-L.
  $${\overset{\wedge }{O}}_{t+1}=\left(\frac{1}{1-x}\right){\left(\frac{S_{t+1}}{K}\right)}^{1/m}-\left(\frac{x}{1-x}\right)I_{t}$$ (15)
  $${\overset{\wedge }{O}}_{t+1}=\left(\frac{1}{1-x}\right){\left(\frac{S_{t+1}}{K}\right)}^{1/m}-\left[\frac{x\left(1+\alpha \right)}{1-x}\right]I_{t}$$ (16)
  Note that Eqs (15) and (16) use the observed inflow at the previous time-point (*I*_*t*_) instead of the observed inflow at the current time (*I*_t+1_) compared with Eqs (7) and (11) because Eq (15) occasionally provides better estimated outflow $\left({\overset{\wedge }{O}}_{t+1}\right)$ as suggested and reported by [6, 8].
**Step 6**: Repeat Steps 2–5 for all time steps.

## Two Improved Heuristic Algorithms

### Elitist-mutated PSO

#### Standard PSO and Its Developments

The PSO algorithm, originally introduced by Kennedy and Eberhart [22], is a population-based stochastic search technique inspired by the social behavior of fish schooling or bird flocking. In PSO, each individual within the swarm is called as a particle and represents a candidate solution to the optimization problem. For a *D*-dimensional search space, assume that *X*_*i*_ = (*x*_i1_, *x*_i2_, …, *x*_*iD*_) and *V*_*i*_ = (*v*_i1_, *v*_i2_, …, *v*_*iD*_) denote the position vector and the velocity vector of the *i*th particle, respectively. The best previously visited position of the *i*th particle and the current global best position in the swarm are recorded as pbest_i_ = (*p*_i1_, *p*_i2_, …, *p*_*iD*_) and gbest = (*p*_g1_, *p*_g2_, …, *p*_*gD*_), respectively, where *g* is the index of the best particle in the swarm. During iterations, the swarm is manipulated according to the updating rules written as Eqs (17) and(18). Note that such process involves individual intelligence, i.e., the particles learn through their own experience (local search) and the experience of their peers (global search).

$$v_{id}^{n+1}=v_{id}^{n}+c_{1}r_{1}\left(p_{id}^{n}-x_{id}^{n}\right)+c_{2}r_{2}\left(p_{gd}^{n}-x_{id}^{n}\right)$$ (17)

$$x_{id}^{n+1}=x_{id}^{n}+v_{id}^{n+1}$$ (18)

where *d* = 1, …, *D* represents the index for the decision variables; *i* = 1, …, pop and pop = the number of particles in the swarm; *n* = iteration number; *r*_1_ and *r*_2_ = uniformly generated random numbers in [0, 1]; *c*_1_ and *c*_2_ = cognitive and social parameters, respectively, which are referred to as acceleration constants. In addition, the value of velocity $v_{id}^{n}$ in each iteration should be limited within the range [−*v*_*d*,*max*_, *v*_*d*,*max*_], where $v_{d,\text{max}}=\upsilon \times \left(x_{d}^{u}-x_{d}^{l}\right)$, 0.05≤ *υ ≤0*.*50*, $x_{d}^{u}$ and $x_{d}^{l}$ are the lower bound and upper bound of the dimension *d*.

Eqs (17) and (18) yield the standard PSO that may have shortcomings of premature convergence and poor control of its search capability. To overcome these drawbacks, extended studies and developments were reported [23–26]. Among which, two big variations focus on modifying the model coefficients are as follows.

In 1988, Shi and Eberhart [26] introduced a linearly decreasing inertia weight parameter *w*^*n*^ into Eq (17) to balance the local search and the global search abilities, which are expressed as Eqs (19) and (20)

$$v_{id}^{n+1}=w^{n}v_{id}^{n}+c_{1}r_{1}\left(p_{id}^{n}-x_{id}^{n}\right)+c_{2}r_{2}\left(p_{gd}^{n}-x_{id}^{n}\right)$$ (19)

$$w^{n}=w_{\text{max}}-\left(w_{\text{max}}-w_{\text{min}}\right)\times \frac{n}{\text{N}}$$ (20)

where *w*_max_ and *w*_min_ = the initial and the final inertia weights, respectively; *n* = current iteration number; and N = maximum iterations.

In 1999, Clerc [23] introduced the constriction factor χ to control the changes in velocity and assure better convergence of the PSO, as shown in Eqs (21) and (22).

$$v_{id}^{n+1}=\chi \left[v_{id}^{n}+c_{1}r_{1}\left(p_{id}^{n}-x_{id}^{n}\right)+c_{2}r_{2}\left(p_{gd}^{n}-x_{id}^{n}\right)\right]$$ (21)

$$\begin{array}{ccc} \chi =\frac{2}{\left|2-\varphi -\sqrt{\varphi^{2}-4\varphi }\right|}, & \text{where}\;\;\;\;\varphi =c_{1}+c_{2}, & \varphi >4 \end{array}$$ (22)

Other developments on improving the performance of PSO fall into two categories [24]: (1) considering the population structure [25, 27] and (2) altering the interaction modes between each particle and its neighbors [24, 28–30].

#### Latest Velocity Updating Rule and Elitist Mutation Operator

**Fig 1:**
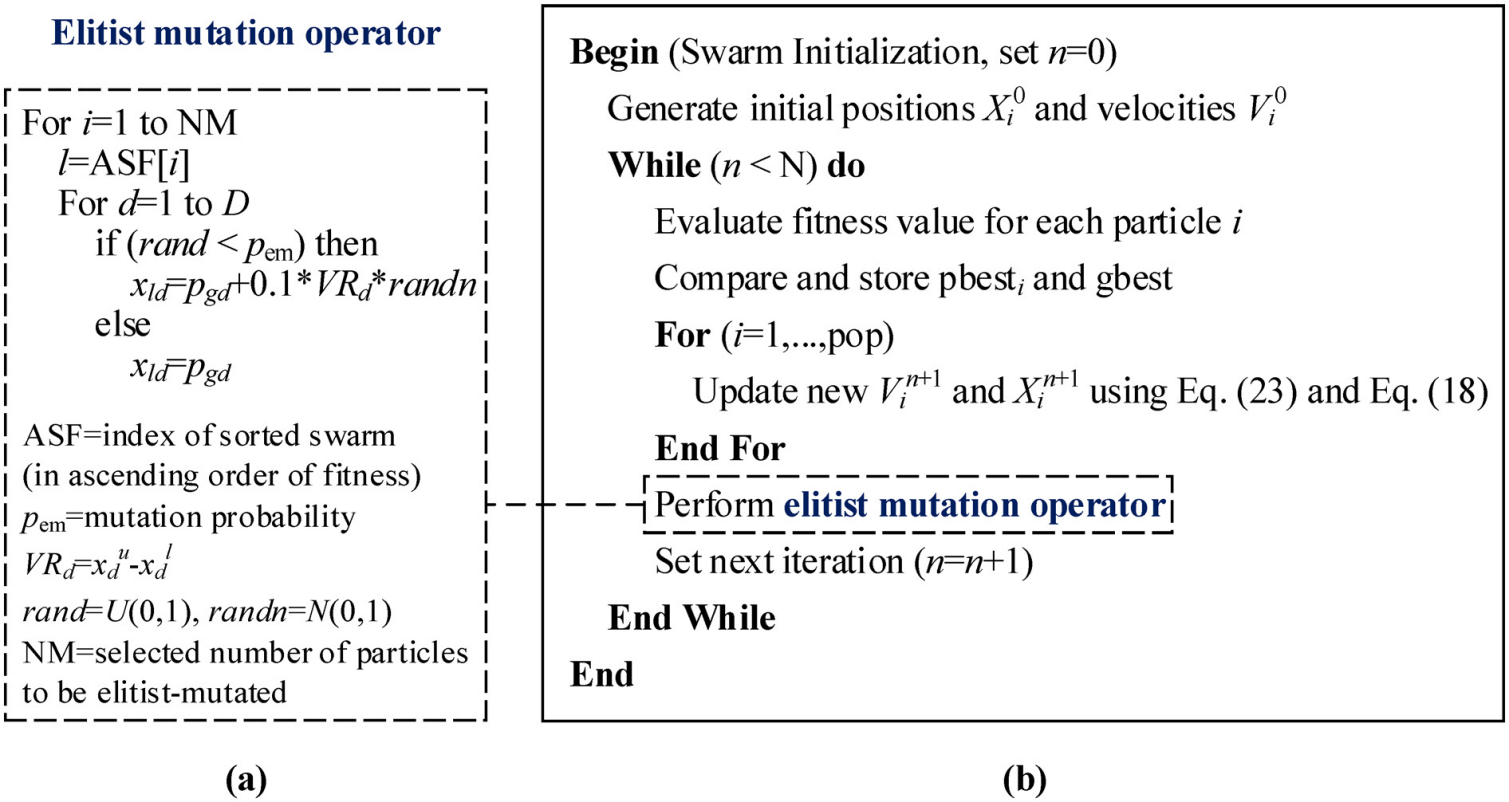
Pseudo-code of the EMPSO algorithm.

A newly developed PSO, namely, elitist-mutated PSO (EMPSO), was proposed by Nagesh Kumar and Janga Reddy [19] for solving water resource problems. Two main improvements in the EMPSO algorithm are: (1) each particle calculates its velocity using the latest updating rule as shown in Eq (23), where *χ* is the constriction coefficient and *w* is the inertia weight; and (2) a new strategic mechanism called elitist mutation operator is introduced to enhance the diversity of the swarm and explore new regions in the whole search space. Pseudo-code of the EMPSO algorithm is presented in Fig 1, in which Fig 1a gives the implementation of the elitist mutation operator and Fig 1b describes the main steps involved in the EMPSO methodology.

$$v_{id}^{n+1}=\chi \left[wv_{id}^{n}+c_{1}r_{1}\left(p_{id}^{n}-x_{id}^{n}\right)+c_{2}r_{2}\left(p_{gd}^{n}-x_{id}^{n}\right)\right]$$ (23)

As illustrated in Fig 1a, in each iteration, the elitist mutation operator is performed on a predefined number (NM) of the worst fitness particles in the swarm. This process of random perturbation is described as follows: first, all particles are sorted in ascending order based on their fitness function and the index numbers for the respective particles are obtained (i.e. index of the sorted swarm is recorded in ASF[*i*], *i* = 1, …, pop); second, the elitist mutation is performed on the front NM worst particles (selected number of least ranked particles to be elitist-mutated) and the respective particle position vectors are replaced with the new mutated position vectors obtained after performing variable-wise mutation on the global best position vector (*p*_em_ is the mutation probability), whereas the velocity vectors of these particles are unvaried.

### Improved GSA

#### Basic GSA

Based on the law of gravity and mass interactions, in 2009, Rashedi and Nezamabadi-pour [20] proposed a novel heuristic algorithm, namely, the gravitational search algorithm (GSA). For an optimization problem, the searcher agents in GSA are a collection of masses in which the values of the masses are proportional to their fitness functions. During the iterative process, the masses interact with each other according to Newtonian gravity and the laws of motion. A heavier mass has a higher attraction, which indicates greater efficiency (similar to the global optimum) and a slower speed of movement. The basic GSA is mathematically described as follows.

Consider a system with pop agents (masses), in which the position of the *i*th agent (candidate solution) is denoted by

$$\begin{array}{cc} X_{i}=\left(x_{i}^{1},\ldots ,x_{i}^{d},\ldots ,x_{i}^{D}\right) & \text{for}\;\;\;\;i=1,\ldots ,\text{pop} \end{array}$$ (24)

where $x_{i}^{d}$ represents the position of the *i*th agent in the *d*th dimension and *D* is the space dimension. According to the Newton law of gravity, the force acting on the *i*th mass from the *j*th mass at time *t* is defined as Eq (25). The total force acting on agent *i* in the dimension *d* is considered to be a randomly weighted sum of the forces exerted from other agents, as expressed in Eq (26)

$$F_{ij}^{d}\left(t\right)=G\left(t\right)\frac{M_{pi}\left(t\right)\times M_{aj}\left(t\right)}{R_{ij}\left(t\right)+\varepsilon }\left(x_{j}^{d}\left(t\right)-x_{i}^{d}\left(t\right)\right)$$ (25)

$$F_{i}^{d}\left(t\right)=\sum_{j\in Kbest,j\neq i}rand_{j}F_{ij}^{d}\left(t\right)$$ (26)

where *M*_*pi*_, *M*_*aj*_ = the passive and active gravitational masses of agent *i* and agent *j*, respectively; *M*_*ai*_ = *M*_*pi*_ = *M*_*i*_ *i* = 1, …, pop, *M*_*i*_ is the inertial mass of the *i*th agent; *G*(*t*) = gravitational constant at time *t*; *ε* = a small constant; *R*_*ij*_(*t*) = ||*X*_*i*_(*t*), *X*_*j*_(*t*)||_2_, is the Euclidian distance between agents *i* and *j*; *rand*_*j*_ = a uniformly generated random number in [0, 1]; *Kbest* = the set of first *K* agents with the best fitness values and the largest masses, which is a function of time *t*, has the initial value *K*_0_ = pop and is linearly decreases to 1 at the end of each iteration.

Note that the gravitational constant *G* is a function of the initial value (*G*_0_, a problem-dependent parameter) and time *t*:

$$G\left(t\right)=G\left(G_{0},t\right)=G_{0}\text{exp}\left(-\beta \times \frac{t}{\text{N}}\right)$$ (27)

Based on the law of motion, the acceleration of the *i*th agent in the *d*th dimension at time *t* is given by

$$a_{i}^{d}\left(t\right)=\frac{F_{i}^{d}\left(t\right)}{M_{i}\left(t\right)}$$ (28)

The next velocity of each agent *i* is considered to be a fraction of its current velocity added to its acceleration and is expressed as follows:

$$v_{i}^{d}\left(t+1\right)=rand_{i}\times v_{i}^{d}\left(t\right)+a_{i}^{d}\left(t\right)$$ (29)

$$x_{i}^{d}\left(t+1\right)=x_{i}^{d}\left(t\right)+v_{i}^{d}\left(t+1\right)$$ (30)

The inertia mass values of the masses are calculated by

$$\begin{array}{ccc} m_{i}\left(t\right)=\frac{fit_{i}\left(t\right)-worst\left(t\right)}{best\left(t\right)-worst\left(t\right)} & \text{and} & M_{i}\left(t\right)=\frac{m_{i}\left(t\right)}{\sum_{j=1}^{N}m_{j}\left(t\right)} \end{array}$$ (31)

where *fit*_*i*_(*t*) = the fitness value of the agent *i* at time *t*; *worst*(*t*) and *best*(*t*) = the worst fitness and the best fitness, respectively, among all agents.

#### Modified Velocity Updating Rule and Elite Strategy

**Fig 2:**
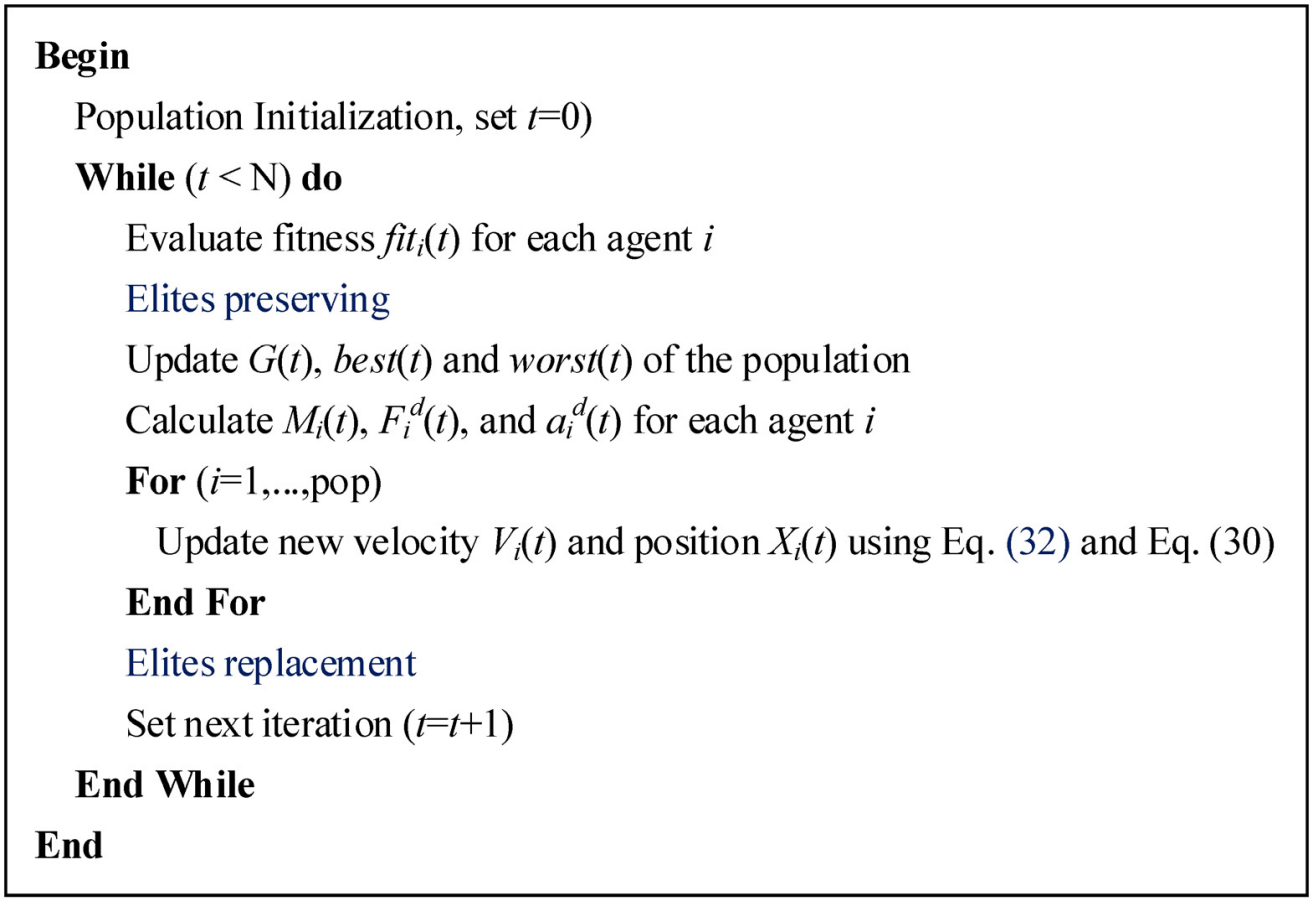
Pseudo-code of the IGSA.

The basic GSA may spend a significant amount of time converging to the global optimum due to the presence of heavier masses at the end of every run. Therefore, we propose an improved GSA (IGSA) which employs the following two strategies to overcome this drawback. The first strategy learns from the idea of memory and social information of PSO and defines a new velocity updating rule for agents, which is written as Eq (32) [31]. The second strategy adds the elite strategy to GSA to accelerate its convergence speed. The idea is to directly preserve a certain number of elite agents in the current generation and replace an equal number of worst agents of the new generated offspring generation. The top 5% of agents are preserved in each generation. Pseudo-code of the IGSA is shown in Fig 2.

$$v_{i}^{d}\left(t+1\right)=rand_{i}\times v_{i}^{d}\left(t\right)+c_{1}r_{1}a_{i}^{d}\left(t\right)+c_{2}r_{2}\left(x_{g}^{d}\left(t\right)-x_{i}^{d}\left(t\right)\right)$$ (32)

where *rand*_*i*_, *r*_1_ and *r*_2_ = uniformly generated random numbers in [0, 1]; *c*_1_ and *c*_2_ = weighting factors; and $x_{g}^{d}(t)$ = the current best solution.

### Performance Test Using Benchmark Functions

Benchmark functions are commonly recognized as an important tool to validate the performance of optimization algorithms [24, 25, 32]. There have been many kinds of benchmark functions reported in the literature [33, 34]. However, only a comprehensive selection of benchmark functions with various characteristics can be truly useful to test new algorithms in an unbiased way. For this reason, a rich test suite of 9 standard minimization benchmark functions with diverse properties in terms of separability, modality were used as experiments for evaluating the EMPSO and IGSA. Table 1 gives a detailed description of these functions, where *D* is the dimension of the function, *f*_min_ is the optimum value of the function. Functions f1–f7 are high-dimensional problems. The first four functions (f1 to f4) are unimodal, which are relatively easy to solve. Functions f5–f9 are multimodal so that the algorithm really suffers from being premature. Functions f5–f7, where the number of local minima increases exponentially with the problem dimension, appear to be the most difficult class of optimization problems. Functions f8 and f9 are two low-dimensional functions which have only a few local minima. We applied the EMPSO and IGSA to the above 9 benchmark functions and compared the experimental results with those obtained by the standard PSO as well as basic GSA. The results are averaged over 50 independent runs and the best-so-far solution, mean and standard deviation of the best solution in each run are listed in Table 2. In all cases, population size is set to 50 (pop = 50) and maximum number of iterations is 1000 (N = 1000). For PSO and EMPSO, the acceleration constants are *c*_1_ = 1.0 and *c*_2_ = 0.5. Other parameters of the EMPSO use the following settings: constriction factor *χ* = 0.9, inertia weight *w* = 1.0, mutation probability *p*_em_ = 0.2, and the size of the elitist-mutated particles NM = 10. For GSA and IGSA, *G*_0_ = 1.0 and *β* = 20.0, whereas *c*_1_ = 0.5 and *c*_2_ = 1.5 for the IGSA.

**Table 1:** Benchmark functions.

No.	Formula	*D*	Range	*f*_mim_	Separability	Modality
f1	$f_{1}\left(X\right)=\sum_{i=1}^{D}x_{i}^{2}$	30	[–100, 100]^*D*^	0	Separable	Unimodal
f2	$f_{2}\left(X\right)=\sum_{i=1}^{D}\left|x_{i}\right|+\prod_{i=1}^{D}\left|x_{i}\right|$	30	[–10, 10]^*D*^	0	Non-Separable	Unimodal
f3	$f_{3}\left(X\right)=\sum_{i=1}^{D}{\left(\sum_{j=1}^{i}x_{j}\right)}^{2}$	30	[–100, 100]^*D*^	0	Non-Separable	Unimodal
f4	$f_{4}\left(X\right)=\sum_{i=1}^{D-1}\left[100{\left(x_{i+1}-x_{i}^{2}\right)}^{2}+{\left(x_{i}-1\right)}^{2}\right]$	30	[–30, 30]^*D*^	0	Non-Separable	Unimodal
f5	$f_{5}\left(X\right)=\sum_{i=1}^{D}-x_{i}\text{sin}\left(\sqrt{\left|x_{i}\right|}\right)$	30	[–500, 500]^*D*^	-418.9829×*D*	Separable	Multimodal
f6	$f_{6}\left(X\right)=-20\;\text{exp}\left(-0.2\sqrt{\frac{1}{D}\sum_{i=1}^{D}x_{i}^{2}}\right)-\text{exp}\left(\frac{1}{D}\sum_{i=1}^{D}\text{cos}\left(2\pi x_{i}\right)\right)+20+e$	30	[–32, 32]^*D*^	0	Non-Separable	Multimodal
f7	$f_{7}\left(X\right)=\frac{1}{4000}\sum_{i=1}^{D}x_{i}^{2}-\prod_{i=1}^{D}\text{cos}\left(\frac{x_{i}}{\sqrt{i}}\right)+1$	30	[–600, 600]^*D*^	0	Non-Separable	Multimodal
f8	$f_{8}\left(X\right)={\left(\frac{1}{500}+\sum_{j=1}^{25}\frac{1}{j+\sum_{i=1}^{2}{\left(x_{i}-a_{ij}\right)}^{6}}\right)}^{-1}$	2	[-65.536, 65.536]^*D*^	1	Non-Separable	Multimodal
f9	$f_{9}\left(X\right)=-\sum_{i=1}^{10}{\left[\left(X-a_{i}\right){\left(X-a_{i}\right)}^{T}+c_{i}\right]}^{-1}$	4	[0, 10]^*D*^	-10.5	Non-Separable	Multimodal

The values of *a*_*ij*_ in f8 are given in S1 Table.

The vectors *a*_*i*_ and *c*_*i*_ in f9 are given in S2 Table.

**Table 2:** Minimization results of benchmark functions in Table 1.

Function	Statistics	PSO	EMPSO	GSA	IGSA
f1	Best	3.03E+03	9.46E-16	8.94E-18	**1.43E-18**
	Mean	7.45E+03	1.29E-05	1.98E-17	**3.48E-18**
	Std.	1.99E+03	8.8E-05	5.74E-18	**9.70E-19**
f2	Best	2.93E+01	1.73E-02	1.32E-08	**5.43E-09**
	Mean	8.40E+01	2.80E-01	2.30E-08	**7.72E-09**
	Std.	6.46E+01	2.12E-01	3.60E-09	**1.33E-09**
f3	Best	1.95E+04	**7.97**	5.77E+04	1.48E+02
	Mean	3.06E+04	**7.13E+01**	1.02E+05	2.54E+03
	Std.	5.98E+03	**6.01E+01**	2.95E+04	1.66E+03
f4	Best	1.81E+06	1.78E+01	2.57E+01	**1.45E+01**
	Mean	6.85E+06	6.76E+01	**2.69E+01**	4.96E+01
	Std.	3.17E+06	6.68E+01	**5.29**	4.21E+01
f5	Best	-9091.9	**-11437.2**	-4249.3	-9299.7
	Mean	-7273.4	**-10205.5**	-2907.9	-7604.1
	Std.	8.23E+02	**4.51E+02**	4.67E+02	6.26E+02
f6	Best	1.26E+01	9.31E-01	2.43E-09	**1.07E-09**
	Mean	1.45E+01	2.00	**3.35E-09**	8.45E-01
	Std.	1.05	4.79E-01	**4.55E-10**	1.27
f7	Best	3.43E+01	**3.83E-14**	1.25	4.72E-02
	Mean	6.86E+01	**3.59E-02**	4.10	1.61
	Std.	1.91E+01	**4.38E-02**	1.62	2.08
f8	Best	**0.9980**	**0.9980**	**0.9980**	**0.9980**
	Mean	0.9981	**0.9980**	3.4961	1.2553
	Std.	1.74E-04	**3.33E-16**	2.25	8.58E-01
f9	Best	-10.0931	**-10.5364**	**-10.5364**	**-10.5364**
	Mean	-6.6513	-5.5079	-9.3011	**-10.5364**
	Std.	1.53	3.58	2.83	**8.88E-15**

Best: best-so-far solution over 50 runs.

Mean: mean of the best solutions in 50 runs.

Std.: standard deviation of the best solutions in 50 runs.

As can be seen from Table 2, the best results are indicated in bold font. Generally speaking, the EMPSO provides much better results than PSO for all the 9 benchmark functions according to the three statistics (Best, Mean and Std.). The IGSA can find better solutions than GSA for functions f1–f7 and it strikingly improves the robustness of GSA (smaller values of Std.) on all functions except for function f6. If comprehensively consider the values of Best and Std., the EMPSO performs the best on 5 functions (f1, f2, f4, f6, f9) and IGSA is the best on the other 4 functions (f3, f5, f7, f8). The results in Table 2 also show that EMPSO and IGSA have better global optimization abilities than the PSO and GSA in solving most of the 9 benchmark functions and can obtain similar solutions.

## Numerical Cases

In the parameter optimization problems of Muskingum models, minimization of the sum of the squared deviations (SSQ) or the sum of the absolute deviations (SAD) between the observed and the estimated outflows is always adopted as the objective function *f*, defined as follows:

$$\begin{array}{cc} \text{Minimize:} & f=\text{SSQ}=\sum_{t=1}^{T}{\left[O_{t}-{\overset{\wedge }{O}}_{t}\left(P\right)\right]}^{2} \end{array}$$ (33)

$$\begin{array}{cc} \text{Minimize:} & f=\text{SAD}=\sum_{t=1}^{T}\left|O_{t}-{\overset{\wedge }{O}}_{t}\left(P\right)\right| \end{array}$$ (34)

where *O*_*t*_ = observed outflow at time *t*; ${\overset{\wedge }{O}}_{t}\left(P\right)$ = estimated outflow at time *t* by the Muskingum routing equation that is Eq (4) for the LMM, Eq (15) for the NLMM and Eq (16) for the NLMM-L; *P* = the parameter vector need to be calibrated, where *P* = (*C*_0_, *C*_1_) in LMM, *P* = (*K*, *x*, *m*) in NLMM, *P* = (*K*, *x*, *m*, *α*) in NLMM-L.

To evaluate the practicability of the EMPSO algorithm and the IGSA in engineering applications, we applied these two improved heuristic algorithms to seek the optimal parameter vector *P* for the three different Muskingum models and compared the results with those obtained by RGA and standard PSO, as well as the basic GSA. The optimal parameter vectors obtained in this study are also compared with the best existing solutions reported in previous literature. For the above five algorithms, the iterations proceed until the stopping criterion is satisfied, which is expressed as

$$\begin{array}{ccc} \left|f_{\text{best}}\left(n\right)-f_{\text{best}}\left(n-1\right)\right|\leq \delta & \text{or} & n\leq \text{N} \end{array}$$ (35)

where *n* is the iteration number and N is the maximum number of iterations (set to 5000); *f*_best_(*n*) is the best value of *f* in the *n*th iteration and *δ* is convergence accuracy.

For the five algorithms in applications, the population size pop was set to 50 and they were implemented on a PC with a 32-bit Windows 7 operating system, 4 GB RAM and 2.93 GHz-core (TM) i3-based processor. Each algorithm was performed over 50 runs on the three Muskingum models for the numerical examples. In RGA, the tournament selection, simulated binary crossover (SBX) and polynomial mutation operators [35] are used; the crossover probability *p*_*c*_ = 0.85 and mutation probability *p*_*m*_ = 0.05; the distribution index for SBX is 10 and the distribution index for the mutation operator is 100. Parameter settings of the PSO, EMPSO, GSA and IGSA are the same with Sect. 3.

### Case 1: Application to LMM

#### Flood Data from the South Canal of China in August 1961

A flood occurred in the south canal of China between the Linqing River and the Chenggou Bay in August 1961, in which the inflow and outflow hydrographs exhibit obvious linear characteristics [36], is employed as the numerical case for the LMM, where Δ*t* = 12*h* and *T* = 28. The search ranges for the two parameters in LMM are set to *C*_0_, *C*_1_ ∈ (0.00, 0.50). One best existing solution according to the literature [11] is *C*_0_ = 0.4736, *C*_1_ = 0.0301 and SAD = 141.225, which is used as a reference.

#### Results and Analysis

**Fig 3:**
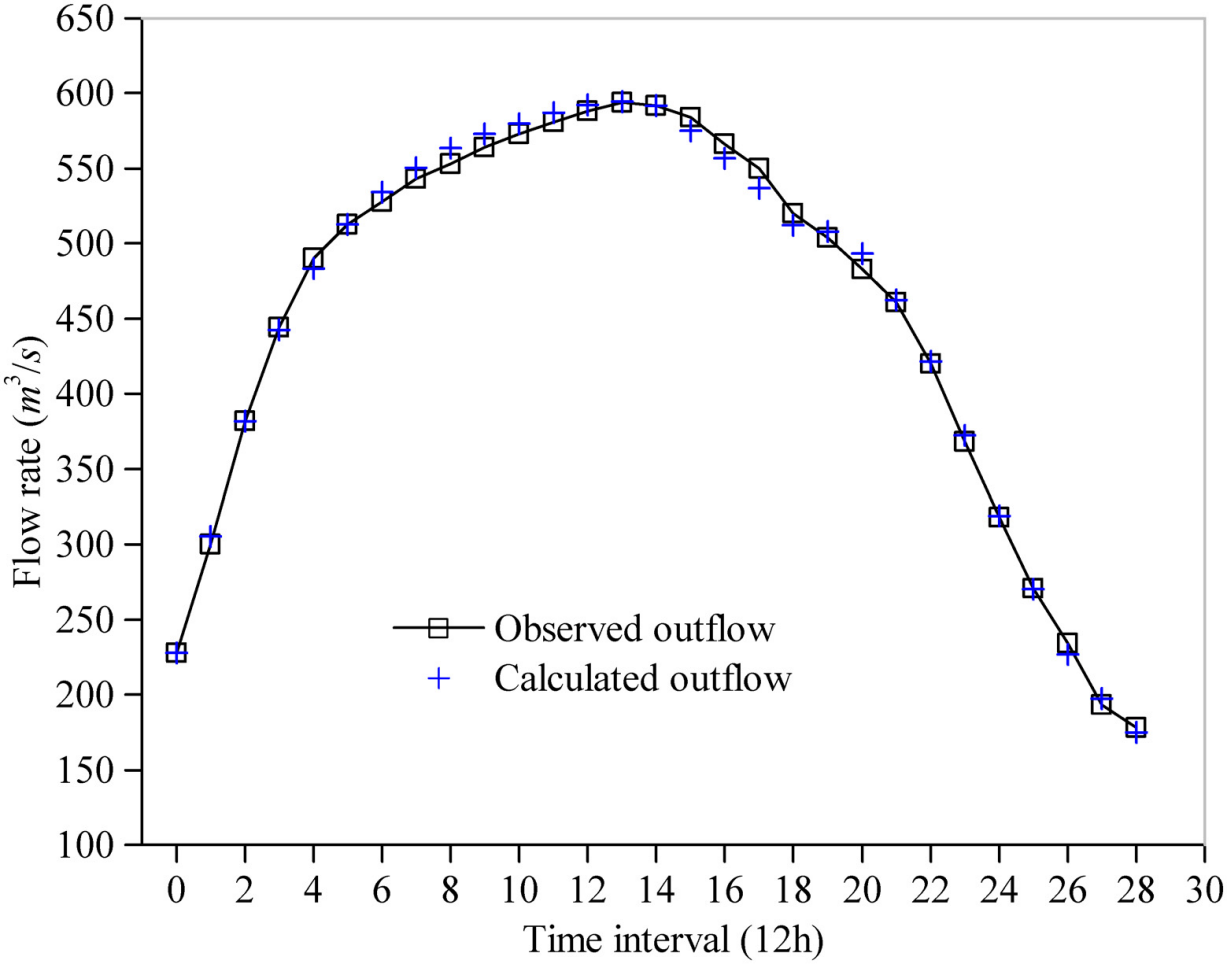
Fitting curve of outflow hydrograph of the LMM experiment.

**Fig 4:**
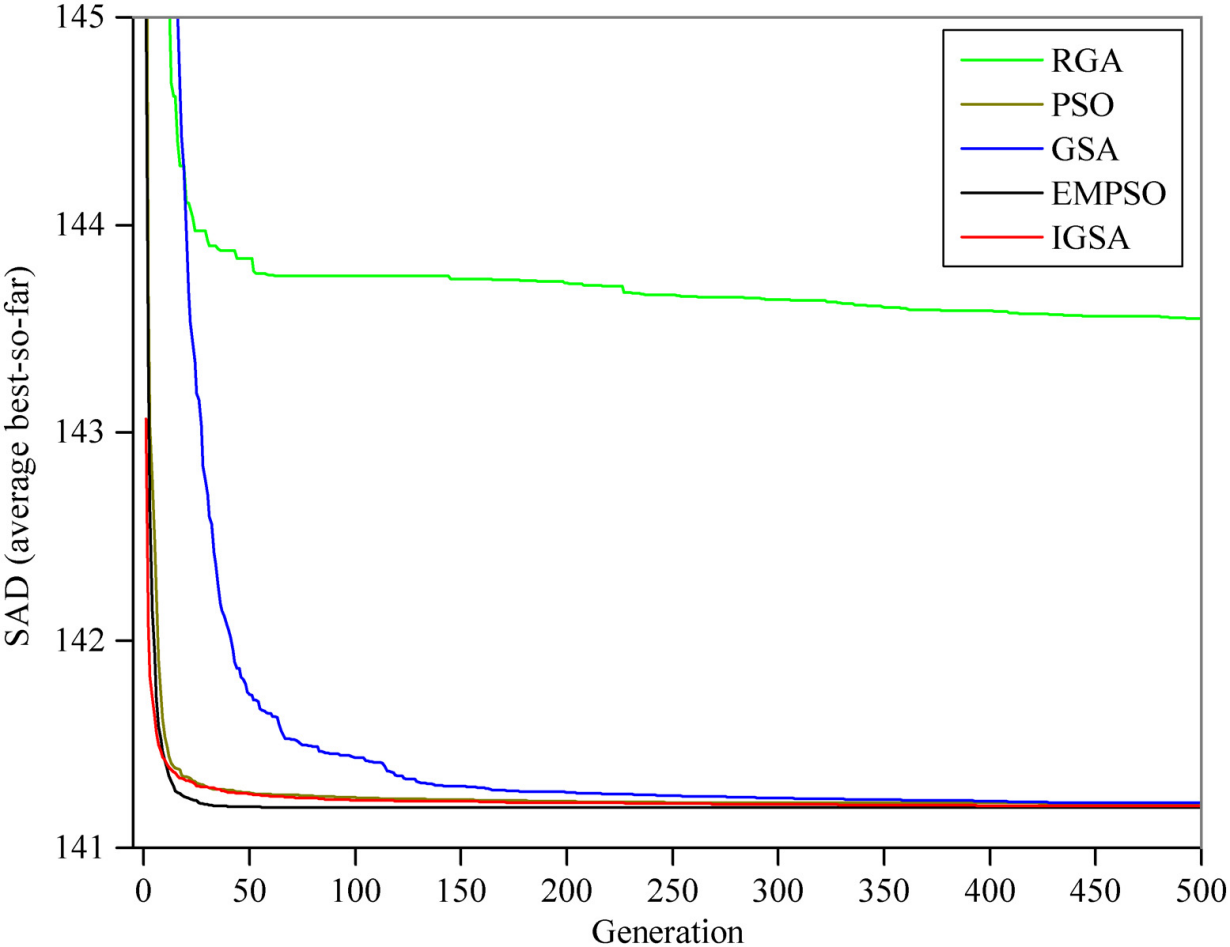
Average best curves for the LMM. All results represent the means of the 50 runs.

For comparison, the statistical results (Best, Worst, Mean, and Std.) of the SAD, the model parameters, the iteration number and the CPU time for convergence (convergence accuracy *δ*) by the five algorithms (RGA, PSO, GSA, EMPSO, IGSA) are listed in Table 3: (1) with the exception of RGA, the other four algorithms find the same optimal solution (SAD = 141.194; *C*_0_ = 0.4729 and *C*_0_ = 0.0317) after 50 runs, which is better than the reference; however, only the GSA, EMPSO and IGSA can steadily converge to the same optimal solution for the LMM in every run (values of Std. for the SADs and the parameters are 0.0000E+00), whereas the optimal solutions obtained by the PSO are slightly fluctuate between different runs; (2) the GSA and IGSA require more time to converge than other three algorithms; (3) compared with GSA and IGSA, the EMPSO has a faster convergence speed (only requires 120 iterations and an average 0.0067s of CPU time for convergence in every run) and exhibits better stability (smallest values of Std.). The estimated outflow hydrograph by the LMM using the best parameter vector obtained in this study is shown in Fig 3. Fig 4 shows the comparison of the average convergence rate among the five algorithms on the LMM.

**Table 3:** Statistics of different algorithms performed on the LMM over 50 runs for the 1961 flood from the south canal of China.

Algorithms	Statistics	*f*	*P*		Convergence (δ = 0.001)	
		SAD	*C*_0_	*C*_1_	Iterations	CPU (s)
RGA	Best	141.196	0.4729	0.0316	4367	0.3852
	Worst	141.301	0.4721	0.0327	4491	0.3977
	Mean	141.220	0.4731	0.0312	3260	0.2894
	Std.	2.1616E-02	4.8277E-04	1.0421E-03	992.01	8.6446E-02
PSO	Best	141.194	0.4729	0.0317	2784	0.0668
	Worst	141.208	0.4730	0.0315	1111	0.0277
	Mean	141.200	0.4729	0.0316	2431	0.0586
	Std.	3.1968E-03	7.0425E-05	1.4650E-04	1410.09	3.3789E-02
GSA	Best	141.194	0.4729	0.0317	2147	0.5549
	Worst	141.194	0.4729	0.0317	2813	0.7099
	Mean	141.194	0.4729	0.0317	2519	0.6291
	Std.	**0.0000E+00**	**0.0000E+00**	**0.0000E+00**	**153.99**	**3.1552E-02**
EMPSO	Best	141.194	0.4729	0.0317	50	0.0037
	Worst	141.194	0.4729	0.0317	165	0.0088
	Mean	141.194	0.4729	0.0317	**120**	**0.0067**
	Std.	**0.0000E+00**	**0.0000E+00**	**0.0000E+00**	**23.54**	**1.1736E-03**
IGSA	Best	141.194	0.4729	0.0317	1175	0.3817
	Worst	141.194	0.4729	0.0317	2053	0.6155
	Mean	141.194	0.4729	0.0317	**1754**	**0.5420**
	Std.	**0.0000E+00**	**0.0000E+00**	**0.0000E+00**	**155.33**	**4.2196E-02**

### Case 2: Application to NLMM

#### Data Set of Wilson (1974)

The data set from ref. [37], which had been demonstrated to have a nonlinear relationship between the storage and the weighted-flow [7], is taken as the numerical case for the NLMM. It is a single peak hydrograph that has been previously investigated by many researchers [2, 4, 7, 8, 10, 16–18, 21, 38], where Δ*t* = 6*h* and *T* = 21. The NLMM has three parameters and their search ranges are set to *K* ∈ [0.01, 1.00], *x* ∈ [0.00, 0.30]and *m* ∈ [1.00, 3.00]. One best existing solution for the NLMM refers to Xu and Qiu [17] using the differential evolution (DE) algorithm was *K* = 0.5175, *x* = 0.2869, *m* = 1.8680 and SSQ = 36.77.

#### Results and Analysis

**Fig 5:**
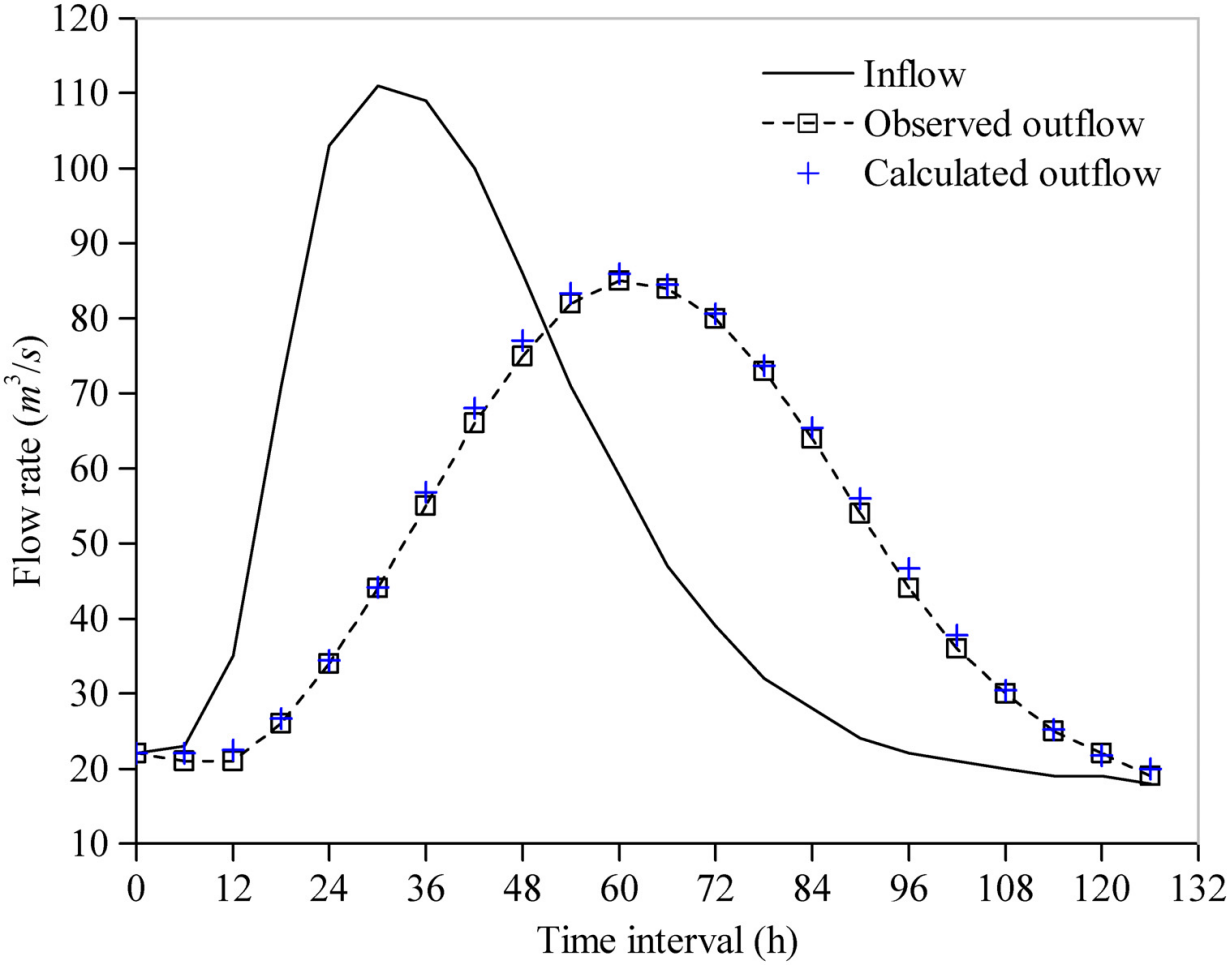
Fitting curve of outflow hydrograph of the NLMM experiment.

**Fig 6:**
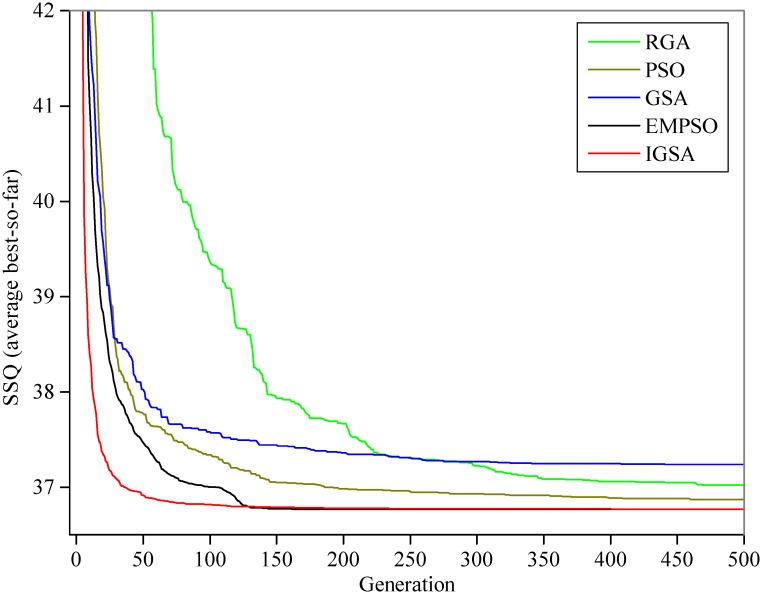
Average best curves for the NLMM.

The statistical results, which resemble Table 3, are also listed in Table 4: (1) only the EMPSO and IGSA can steadily converge to the same optimal solution (SSQ = 36.7679; *K* = 0.5175, *x* = 0.2869 and *m* = 1.8680) for the NLMM in every run, whereas optimal solutions obtained by RGA, PSO and GSA fluctuate between different runs; (2) for GSA, EMPSO and IGSA, the average number of iterations that are required for convergence in every run are approximately 2062, 191 and 780, respectively, and the corresponding CPU consuming times are 0.9992s, 0.0883s and 0.4864s; these data indicate that the EMPSO has a faster convergence speed and the IGSA obviously improves the convergence performance of GSA; (3) the EMPSO has the best performance in optimizing the NLMM (the lowest CPU time for every run and a steady fluctuation among of iterations with the lowest Std. = 51.22). The estimated outflow hydrograph by the NLMM using the best parameter vector obtained in this study is shown in Fig 5. Fig 6 shows the average convergence rate for the five different algorithms on the NLMM.

**Table 4:** Statistics of different algorithms performed on the NLMM over 50 runs for the data set of Wilson (1974).

Algorithms	Statistics	*f*	*P*			Convergence (δ = 0.0001)	
		SSQ	*K*	*x*	*m*	Iterations	CPU (s)
RGA	Best	36.7683	0.5184	0.2868	1.8677	3241	0.9613
	Worst	37.8397	0.6369	0.2881	1.8223	4548	1.3407
	Mean	36.9300	0.5266	0.2873	1.8646	1973	0.5888
	Std.	2.3267E-01	3.3579E-02	1.5334E-03	1.3702E-02	1844.47	5.4068E-01
PSO	Best	36.7691	0.5168	0.2871	1.8684	233	0.0536
	Worst	36.8376	0.5438	0.2875	1.8569	4390	0.9927
	Mean	36.7905	0.5162	0.2869	1.8687	2203	0.5007
	Std.	1.5596E-02	9.3849E-03	6.8361E-04	4.0096E-03	1453.70	3.2952E-01
GSA	Best	36.7694	0.5216	0.2870	1.8663	3022	1.5431
	Worst	38.0759	0.5514	0.2819	1.8555	40	0.0256
	Mean	37.0422	0.5492	0.2871	1.8553	2062	0.9992
	Std.	3.2174E-01	3.3700E-02	1.8361E-03	1.3696E-02	1669.06	7.9225E-01
EMPSO	Best	36.7679	0.5175	0.2869	1.8681	104	0.0475
	Worst	36.7679	0.5175	0.2869	1.8681	321	0.1450
	Mean	36.7679	0.5175	0.2869	1.8681	**191**	**0.0883**
	Std.	**0.0000E+00**	**0.0000E+00**	**0.0000E+00**	**0.0000E+00**	**51.22**	**2.3747E-02**
IGSA	Best	36.7679	0.5175	0.2869	1.8681	366	0.2332
	Worst	36.7679	0.5175	0.2869	1.8681	1051	0.6483
	Mean	36.7679	0.5175	0.2869	1.8681	**780**	**0.4864**
	Std.	**0.0000E+00**	**0.0000E+00**	**0.0000E+00**	**0.0000E+00**	**119.17**	**7.1140E-02**

### Case 2: Application to NLMM-L

#### River Wyre Flood in October 1982

The River Wyre flood event in October 1982 [21], which exhibits a considerable increase of flood volume (lateral flow) between the inflow section and the outflow section (approximately 25km), is selected as the numerical case for the NLMM-L. The flood data have multi-peaked inflow, in which Δ*t* = 1*h* and *T* = 31, and a major lateral flow contribution (which implies a large value of *α*). The search ranges for the four parameters in the NLMM-L are set to *K* ∈ [0.01, 6.00], *x* ∈ [0.00, 0.30], *m* ∈ [0.50, 3.00] and *α* ∈ [0.00, 3.00]. The best existing solution by the cuckoo search (CS) algorithm for the NLMM-L according to the literature [18] is *K* = 5.6765, *x* = 0.2271, *m* = 0.9800, *α* = 2.5298 and SSQ = 53.6574.

#### Results and Analysis

**Fig 7:**
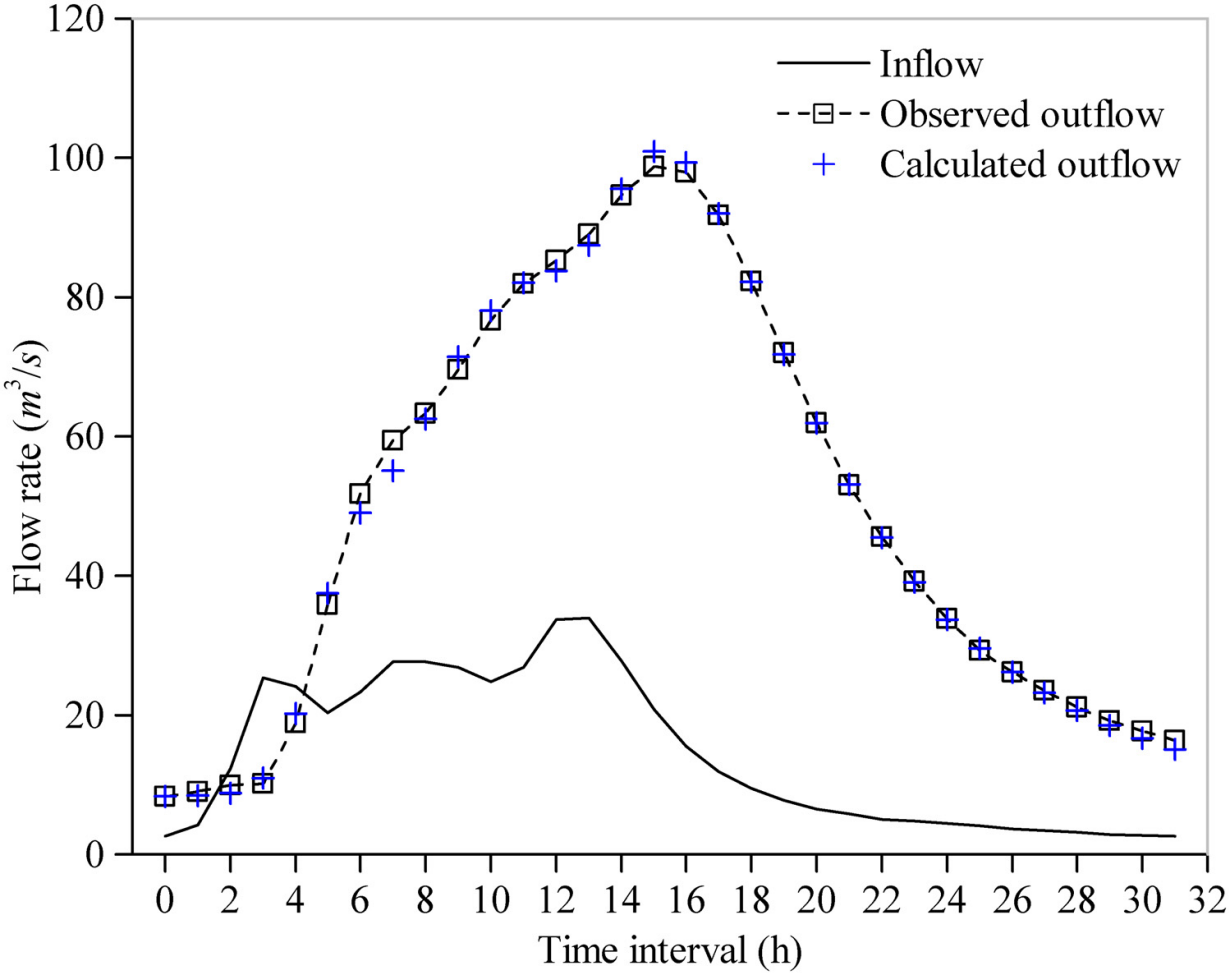
Fitting curve of outflow hydrograph of the NLMM-L experiment.

**Fig 8:**
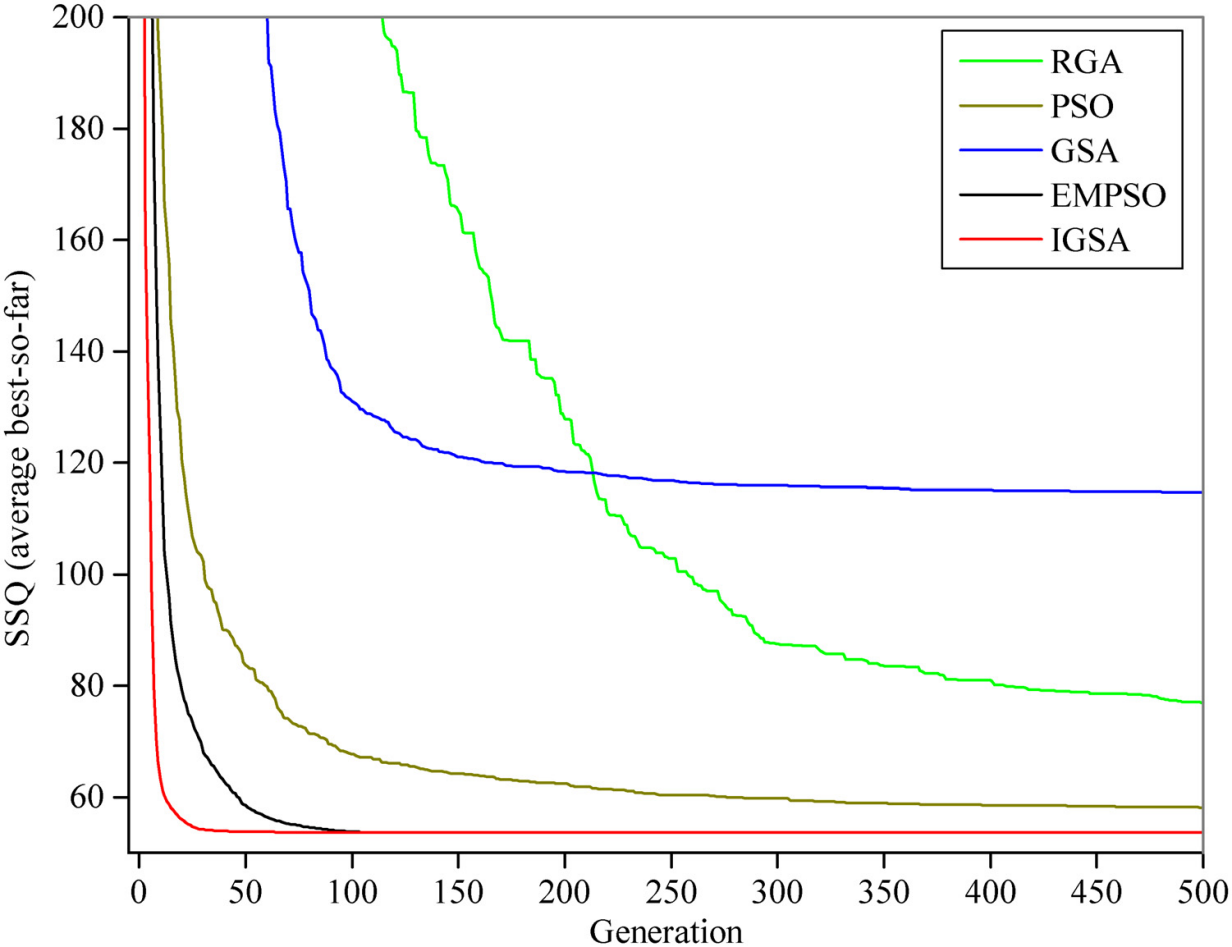
Average best curves for the NLMM-L.

For this numerical case to the NLMM-L, statistical results resemble Table 3 are presented in Table 5. As shown in Table 5: (1) only the EMPSO and IGSA can steadily find the global optimal solution in every run (the values of Std. for the SSQs and the parameters are 0.0000E+00), which is same to the reference; (2) the EMPSO still has the best performance in optimizing the NLMM-L (the lowest average CPU time for every run and a fairly steady fluctuation among iterations with the lowest Std. = 39.82). The estimated outflow hydrograph by the NLMM-L using the best solution obtained in this study is shown in Fig 7. Fig 8 shows the average convergence rate between the five different algorithms on the NLMM-L.

**Table 5:** Statistics of different algorithms performed on the NLMM-L over 50 runs for the River Wyre flood in October 1982.

Algorithms	Statistics	*f*	*P*				Convergence (δ = 0.0001)	
		SSQ	*K*	*x*	*m*	*α*	Iterations	CPU (s)
RGA	Best	53.8173	5.6300	0.2299	0.9821	2.5373	632	0.2863
	Worst	66.9455	4.1086	0.2332	1.0470	2.5230	5000	2.0693
	Mean	58.1929	4.8087	0.2310	1.0150	2.5253	4374	1.8169
	Std.	**3.5486E+00**	3.6741E-01	1.9815E-03	1.6023E-02	2.6082E-03	1285.31	5.2463E-01
PSO	Best	53.7213	5.7644	0.2258	0.9770	2.5319	3195	1.0745
	Worst	56.0369	5.2233	0.2172	0.9960	2.5309	4903	1.6501
	Mean	54.3777	5.6507	0.2277	0.9810	2.5307	2907	0.9797
	Std.	**4.9223E-01**	2.1336E-01	4.6838E-03	7.7166E-03	4.7672E-03	1411.27	4.7516E-01
GSA	Best	67.9146	4.0779	0.2294	1.0474	2.5204	145	0.1187
	Worst	156.8379	2.2746	0.2342	1.1702	2.5148	**4203**	**2.6463**
	Mean	101.8833	3.0891	0.2345	1.1070	2.5183	**4178**	**2.6176**
	Std.	**1.4658E+01**	3.1027E-01	7.9331E-04	2.0579E-02	1.2428E-03	601.50	3.6447E-01
EMPSO	Best	53.6574	5.6765	0.2271	0.9800	2.5298	131	0.0886
	Worst	53.6574	5.6765	0.2271	0.9800	2.5298	298	0.2010
	Mean	53.6574	5.6765	0.2271	0.9800	2.5298	**196**	**0.1330**
	Std.	**0.0000E+00**	**0.0000E+00**	**0.0000E+00**	**0.0000E+00**	**0.0000E+00**	**39.82**	**2.6695E-02**
IGSA	Best	53.6574	5.6765	0.2271	0.9800	2.5298	277	0.2386
	Worst	53.6574	5.6765	0.2271	0.9800	2.5298	780	0.6520
	Mean	53.6574	5.6765	0.2271	0.9800	2.5298	**618**	**0.5201**
	Std.	**0.0000E+00**	**0.0000E+00**	**0.0000E+00**	**0.0000E+00**	**0.0000E+00**	**97.18**	**7.9210E-02**

## Conclusions

In this study, the EMPSO algorithm and the IGSA were applied for solving the parameter estimation problems of three forms of linear or nonlinear Muskingum models (LMM, NLMM and NLMM-L). The LMM has two parameters and the NLMM has three, whereas the NLMM-L considers the lateral flow along the river reach, which has a more complex structure with four parameters. The EMPSO and IGSA were tested on a rich set of 9 standard minimization benchmark functions. Then three typical flood events used in previous literature were selected as numerical cases (Case 1–3) to evaluate the practicability of the EMPSO and IGSA in applications. The results by the EMPSO and IGSA were compared with those obtained by the RGA, PSO and GSA, as well as the best reported solutions in the literature. Several conclusions are summarized as follows.

1. only the EMPSO and IGSA can steadily converge to the same optimal solution for the three Muskingum models in every run compared with RGA, standard PSO and the basic GSA;
2. the GSA may require more iterations and CPU time than the IGSA to find the same optimal solution for the LMM, and the results obtained by the GSA for the NLMM and the NLMM-L are the worst among the five algorithms (the largest values of SSQ and Std. in Tables 4 and 5), which indicates that the proposed IGSA can improve the performance (including the search efficiency, convergence speed and the stability) of GSA in optimizing the NLMM and the NLMM-L;
3. the EMPSO has the fastest convergence rate and the best robustness than the other four algorithms for the three Muskingum models in term of specified convergence accuracy and the Std. values.

## Supporting Information

S1 Table *a*_*ij*_ in function f8. (DOCX)

S2 Table Vectors *a*_*i*_ and *c*_*i*_ in function f9. (DOCX)

S3 Table Original data of each case and optimal estimated outflow hydrographs by the EMPSO and IGSA (*m*^3^/*s*). (DOCX)
