# Supplementary material for: Application of the Elitist-Mutated PSO and an Improved GSA to Estimate Parameters of Linear and Nonlinear Muskingum Flood Routing Models
Source: PLoS One. 2016 Jan 19;11(1):e0147338. doi: 10.1371/journal.pone.0147338 (PMC4718656; doi:10.1371/journal.pone.0147338)
Supplement: S1 Table — (DOCX) [file pone.0147338.s001.docx]

**S1 Table. *a_ij_* in function f8.**

| $\left(a_{ij}\right)=\left(\begin{matrix} -32 & -16 & 0 & 16 & 32 & -32 & \ldots& 0 & 16 & 32 \\ -32 & -32 & -32 & -32 & -32 & -16 & \ldots& 32 & 32 & 32 \end{matrix}\right)$ |
| --- |
