# Supplementary material for: Application of the Elitist-Mutated PSO and an Improved GSA to Estimate Parameters of Linear and Nonlinear Muskingum Flood Routing Models
Source: PLoS One. 2016 Jan 19;11(1):e0147338. doi: 10.1371/journal.pone.0147338 (PMC4718656; doi:10.1371/journal.pone.0147338)
Supplement: S2 Table — (DOCX) [file pone.0147338.s002.docx]

**S2 Table. Vectors *a_i_* and *c_i_* in function f9.**

| *i* | *a_ij_*, *j*=1,…,4 | | | | *c_i_* |
| --- | --- | --- | --- | --- | --- |
| 1 | 4 | 4 | 4 | 4 | 0.1 |
| 2 | 1 | 1 | 1 | 1 | 0.2 |
| 3 | 8 | 8 | 8 | 8 | 0.2 |
| 4 | 6 | 6 | 6 | 6 | 0.4 |
| 5 | 3 | 7 | 3 | 7 | 0.4 |
| 6 | 2 | 9 | 2 | 9 | 0.6 |
| 7 | 5 | 5 | 3 | 3 | 0.3 |
| 8 | 8 | 1 | 8 | 1 | 0.7 |
| 9 | 6 | 2 | 6 | 2 | 0.5 |
| 10 | 7 | 3.6 | 7 | 3.6 | 0.5 |
