# Supplementary material for: Application of the Elitist-Mutated PSO and an Improved GSA to Estimate Parameters of Linear and Nonlinear Muskingum Flood Routing Models
Source: PLoS One. 2016 Jan 19;11(1):e0147338. doi: 10.1371/journal.pone.0147338 (PMC4718656; doi:10.1371/journal.pone.0147338)
Supplement: S3 Table — (DOCX) [file pone.0147338.s003.docx]

**S3 Table. Original data of each case and optimal estimated outflow hydrographs by the EMPSO and IGSA (*m*^3^/*s*).**

| Time | Case 1 (∆*t*=12*h*) | | | Case 2 (∆*t*=6*h*) | | | Case 3 (∆*t*=1*h*) | | |
| --- | --- | --- | --- | --- | --- | --- | --- | --- | --- |
| $t$ | $I_{t}$ | $O_{t}$ | $\mathop{O}\limits^{\wedge} _{t}$ | $I_{t}$ | $O_{t}$ | $\mathop{O}\limits^{\wedge} _{t}$ | $I_{t}$ | $O_{t}$ | $\mathop{O}\limits^{\wedge} _{t}$ |
| 0 | 261 | 228 | 228.00 | 22 | 22 | 22.00 | 2.6 | 8.3 | 8.30 |
| 1 | 389 | 300 | 305.19 | 23 | 21 | 22.00 | 4.2 | 9 | 8.51 |
| 2 | 462 | 382 | 382.00 | 35 | 21 | 22.42 | 12.3 | 9.9 | 8.79 |
| 3 | 505 | 444 | 442.70 | 71 | 26 | 26.61 | 25.4 | 10.2 | 10.94 |
| 4 | 525 | 490 | 483.60 | 103 | 34 | 34.46 | 24.1 | 18.9 | 20.28 |
| 5 | 543 | 513 | 513.00 | 111 | 44 | 44.17 | 20.3 | 35.9 | 37.54 |
| 6 | 556 | 528 | 534.29 | 109 | 55 | 56.85 | 23.3 | 51.8 | 49.07 |
| 7 | 567 | 543 | 550.44 | 100 | 66 | 68.06 | 27.7 | 59.4 | 55.11 |
| 8 | 577 | 553 | 563.53 | 86 | 75 | 77.07 | 27.7 | 63.3 | 62.50 |
| 9 | 583 | 564 | 573.16 | 71 | 82 | 83.32 | 26.9 | 69.6 | 71.44 |
| 10 | 587 | 573 | 580.02 | 59 | 85 | 85.90 | 24.8 | 76.7 | 78.03 |
| 11 | 595 | 581 | 587.32 | 47 | 84 | 84.54 | 26.9 | 82 | 82.07 |
| 12 | 597 | 588 | 592.14 | 39 | 80 | 80.58 | 33.7 | 85.3 | 83.72 |
| 13 | 597 | 594 | 594.59 | 32 | 73 | 73.71 | 33.9 | 89 | 87.43 |
| 14 | 589 | 592 | 592.02 | 28 | 64 | 65.41 | 27.8 | 94.6 | 95.49 |
| 15 | 556 | 584 | 574.89 | 24 | 54 | 56.00 | 20.8 | 98.8 | 100.88 |
| 16 | 538 | 566 | 556.85 | 22 | 44 | 46.67 | 15.6 | 98 | 99.29 |
| 17 | 516 | 550 | 536.93 | 21 | 36 | 37.75 | 11.9 | 91.8 | 92.06 |
| 18 | 486 | 520 | 512.18 | 20 | 30 | 30.47 | 9.5 | 82.3 | 82.22 |
| 19 | 505 | 504 | 507.96 | 19 | 25 | 25.23 | 7.8 | 72 | 71.75 |
| 20 | 477 | 483 | 493.22 | 19 | 22 | 21.74 | 6.5 | 61.9 | 61.91 |
| 21 | 429 | 461 | 462.34 | 18 | 19 | 19.99 | 5.8 | 53 | 53.12 |
| 22 | 379 | 420 | 421.87 |  |  |  | 5 | 45.6 | 45.47 |
| 23 | 320 | 368 | 372.34 |  |  |  | 4.8 | 39.2 | 39.14 |
| 24 | 263 | 318 | 318.97 |  |  |  | 4.5 | 33.8 | 33.76 |
| 25 | 220 | 271 | 270.39 |  |  |  | 4.1 | 29.3 | 29.55 |
| 26 | 182 | 234 | 226.99 |  |  |  | 3.7 | 26.2 | 26.12 |
| 27 | 167 | 193 | 197.20 |  |  |  | 3.4 | 23.5 | 23.20 |
| 28 | 152 | 178 | 174.87 |  |  |  | 3.2 | 21.2 | 20.67 |
| 29 |  |  |  |  |  |  | 2.9 | 19.2 | 18.52 |
| 30 |  |  |  |  |  |  | 2.8 | 17.7 | 16.71 |
| 31 |  |  |  |  |  |  | 2.6 | 16.4 | 15.12 |
| SSQ | 1086.746 | | | **36.768** | | | **53.657** | | |
| SAD | **141.194** | | | 23.466 | | | 28.886 | | |

$I_{t}$=observed inflow at time $t$.

$O_{t}$=observed outflow at time $t$.

$\mathop{O}\limits^{\wedge} _{t}$=estimated outflow at time $t$.
